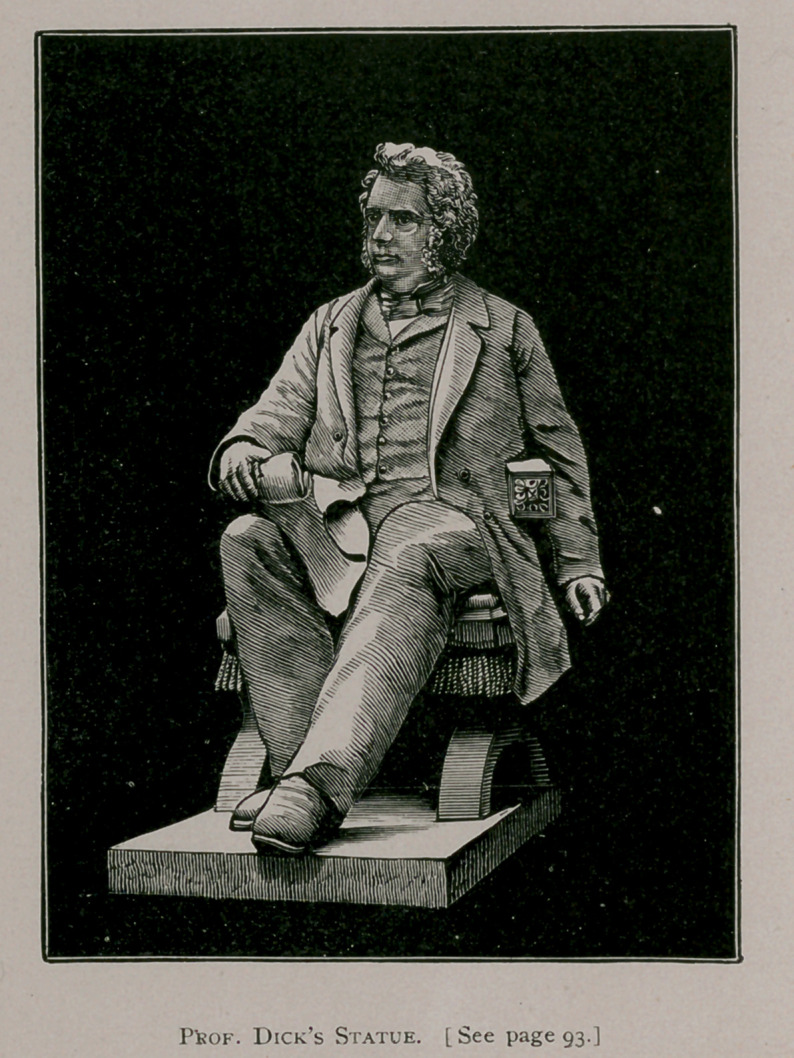# Prof. Dick’s Statue

**Published:** 1884-01

**Authors:** 


Professor Dick's Statue.—The ceremony of the unveiling of this statue was performed by the Lord Provost of Edinburgh, on the 24th of October, in the Dick Royal Veterinary College.

"Professor Dick," the Lord Provost said, "was born in the White-horse Close of the Canongate, ninety years ago. After receiving the ordinary school education he attended lectures at the University on anatomy and chemistry. At that time there was no veterinary college in Scotland. So, having, very naturally—his father being a farrier and smith—conceived a regard for horses and dogs, he went, about 1817, in pursuit of further knowledge of those interesting animals, to London, where there was a veterinary college, and continued there for a year or two, where he got his diploma. In 1820 Professor Dick began his lectures in Edinburgh. In 1837 he received royal recognition through the late Lord Albemarle, then Master of the Horse. From that time onward for the next thirty years Professor's Dick's course was a uniform success, his knowledge, his practice, and his usefulness increasing year by year. During that time he was able, through his industry and talent, to spend £10,000 of his own money in founding this college. He undertook the responsibility of procuring lecturers on all the kindred subjects necessary for obtaining a proper knowledge, of the art, and he left behind him his whole fortune—only subject to the life rent of his venerable sister—for the benefit of this institution. Professor Dick was a strong man, an industrious man, a man who knew the value of work, and a man of whom they had had many types, his lordship was glad to say, in Scotland—one of those men who had done the world a good service.

The Marquis of Lothian, Dr. Fleming, Prof. Walley and others made appropriate remarks in praise of the character of the late Professor and of respect to his memory.

The statue consists of a figure of Professor Dick sitting in the professional chair, holding in his right hand the foot bones of a horse as illustrative of some point on which he is supposed to be earnestly speaking. A most vivid likeness has been produced, the bold and firm modelling of the expressive features and large massive head at once conveying the independence and determination that were so prominent in the character of the man.—*Veterinary Journal.*

## Figures and Tables

**Figure f1:**